# Identification and Classification of Human Body Exercises on Smart Textile Bands by Combining Decision Tree and Convolutional Neural Networks

**DOI:** 10.3390/s23136223

**Published:** 2023-07-07

**Authors:** Bonhak Koo, Ngoc Tram Nguyen, Jooyong Kim

**Affiliations:** 1 Department of Materials Science and Engineering, Soongsil University, Seoul 156-743, Republic of Korea; soongsilkoo@soongsil.ac.kr; 2 Department of Smart Wearable Engineering, Soongsil University, Seoul 156-743, Republic of Korea; tram2310@soongsil.ac.kr

**Keywords:** smart wearables, HAR, exercise classification, cascaded algorithms, IMU, decision tree, 1D-convolution

## Abstract

In recent years, human activity recognition (HAR) has gained significant interest from researchers in the sports and fitness industries. In this study, the authors have proposed a cascaded method including two classifying stages to classify fitness exercises, utilizing a decision tree as the first stage and a one-dimension convolutional neural network as the second stage. The data acquisition was carried out by five participants performing exercises while wearing an inertial measurement unit sensor attached to a wristband on their wrists. However, only data acquired along the *z*-axis of the IMU accelerator was used as input to train and test the proposed model, to simplify the model and optimize the training time while still achieving good performance. To examine the efficiency of the proposed method, the authors compared the performance of the cascaded model and the conventional 1D-CNN model. The obtained results showed an overall improvement in the accuracy of exercise classification by the proposed model, which was approximately 92%, compared to 82.4% for the 1D-CNN model. In addition, the authors suggested and evaluated two methods to optimize the clustering outcome of the first stage in the cascaded model. This research demonstrates that the proposed model, with advantages in terms of training time and computational cost, is able to classify fitness workouts with high performance. Therefore, with further development, it can be applied in various real-time HAR applications.

## 1. Introduction

Human activity recognition (HAR) can be defined as the identification and naming of activities from the raw data of activities acquired by several devices, mostly wearable sensors utilizing artificial intelligence (AI) [1]. Recently, HAR has drawn a lot of attention from researchers due to its critical applications in various domains, including virtual reality (VR), smart homes/offices, sports, healthcare, and robotics [2,3,4,5]. Nowadays, methods to track or monitor human motion can be categorized into observation-based and on-body-sensor-based. In the first group, body movements are captured and recognized by some motion-tracking and depth-sensing devices such as Microsoft Kinect or radar [6,7]. By contrast, on-body sensors directly detect and measure movements based on the characteristics of the sensor itself; for instance, resistive stretch sensors [8] or inertial measurement unit (IMU) sensors [9].

Virtual personal training has become popular in recent years, especially during the pandemic [10,11,12]. In virtual personal training, practitioners can obtain workouts through online platforms but still manage to interact with real trainers or robots. The training systems should be able to determine whether their users are performing the exercises correctly and effectively. At this point, HAR plays an important role in tracking the body movements in each exercise with body-worn sensors. Machine learning is then deployed to learn the features from this data and classify the exercises into the correct groups. Among various types of sensors, IMUs show great potential and have been adopted for training activity recognition [13,14,15,16]. Jay P., et al. utilized wearable IMUs mounted on earphones to detect walking and count steps [10], while Fanuel W. and Fuchun J. researched a similar task, detecting walking exercises, using foot-mounted IMUs and machine learning algorithms [14]. In another study, the authors evaluated the number of IMU sensors for data acquisition and compared four types of classifiers for the task of classifying eight gym exercises [16]. However, these studies presented some limitations on the number of exercises that could be classified. Furthermore, it may not be possible to achieve high accuracy in classifying a large number of exercises if only one algorithm is deployed [17].

Nowadays, the need to track or monitor the motions of users during fitness activities has increased because of its benefits in terms of contribution to the impact of the exercises [18,19,20,21]. Other than that, recognizing and monitoring human motions while the users are performing workouts can help assess the effectiveness of the exercise itself, the training equipment, and the whole training program. To achieve the degree to which a body-worn sensor can acquire and process signals and classify motions in almost real time, the machine learning algorithm embedded in the devices must have not only high accuracy but simplicity, so that it can be implemented quickly and be suitable for wearable applications. One-dimension convolutional neural networks (1D-CNNs) have been utilized to classify time series data [22,23,24] due to their advantages over recurrent neural networks in terms of faster training time and simpler architecture. Therefore, in this study, the authors investigated the performance of a cascaded classification consisting of a decision tree and a 1D-CNN. The database used in this task solely consists of the acceleration data along the z-axis acquired from the IMU sensor, unlike previous methods that had six-axis or three-axis [16,25] inertial sensor data. For comparison, we compared the performance of the proposed method and a 1D-CNN model on the task of classifying fitness exercises and evaluated their efficiencies.

## 2. Materials and Methods

### 2.1. Acceleration Data

**Figure 1:**
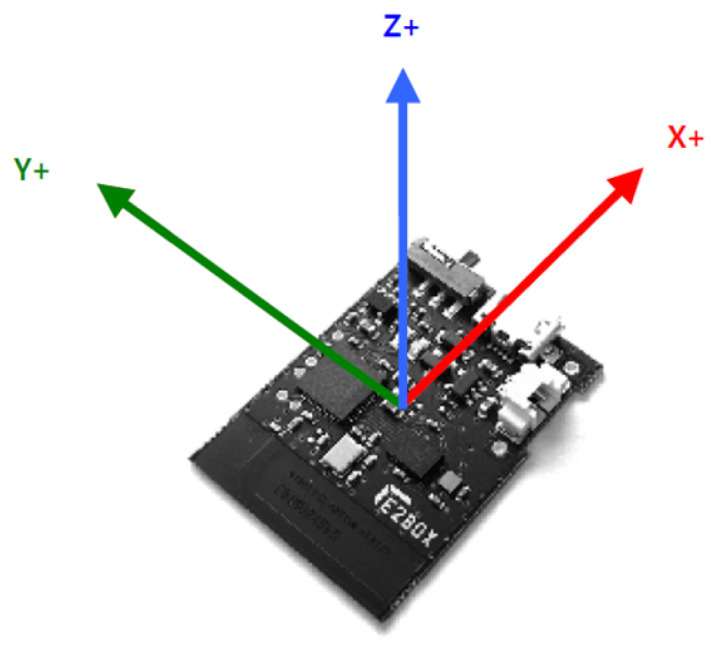
Three axes of the accelerometer on the inertial measurement unit sensor.

IMU sensors use a combination of an accelerometer, a gyroscope, and a magnetometer to measure and report specific forces, angle ratios, and magnetic fields surrounding the body and provide time series data. IMUs can be categorized into 2 groups, which are the 6-axis and the 9-axis. 6-axis IMUs are usually composed of a 3-axis accelerometer and a 3-axis gyroscope, while the 9-axis IMUs include an additional 3-axis magnetometer. In IMUs, the accelerometer measures the linear acceleration along the x-axis, y-axis, and z-axis. In this study, the authors employed EBMotion V5.2, an IMU sensor from E2BOX (Hanam, Gyeonggi, Republic of Korea) which consists of a wireless sensor (EBIMU24GV52) and a wireless receiver (EBRCV24GV5). Figure 1 illustrates the direction of 3 axes of the accelerometer in EBMotion V5.2.

Specifically, the *x*-axis captures the sideways or horizontal movements of the user, while the forward or backward movements are captured by the *y*-axis and the *z*-axis takes responsibility for the upward or downward movements. The accelerometer of the IMU sensor generates three time series along 3 axes, x, y, and z, denoted by A_x_, A_y_, and A_z_, respectively. Each time series is the combination of linear acceleration caused by gravity (g) and body motion (l). However, only A_z_ was utilized for the classification task in this study due to two reasons. Firstly, the study aims to classify upper body exercises based on the user’s forearm postures, which are raising and lowering. Therefore, A_z_ becomes more meaningful, and A_x_ and A_y_ can be neglected. Secondly, the utilization of just A_z_ data considerably helps reduce the computational cost and the amount of data to process.

**Figure 2:**
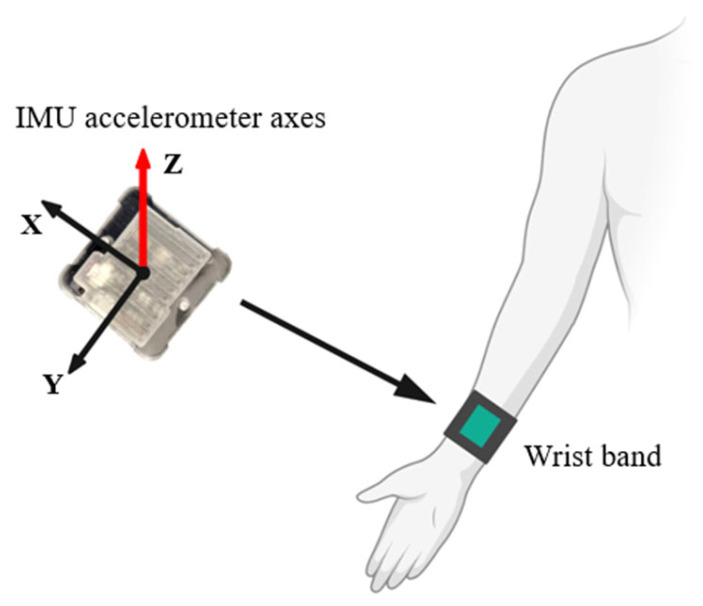
The IMU sensor attachment location on the participant’s wrist.

Figure 2 demonstrates the position of the IMU sensor on the participants’ wrists. For convenience, while they were wearing the wristband, the 3 axes of the accelerometer were swapped from the order of x-y-z to z-x-y so that the *z*-axis of the IMU sensor was always toward the user’s armpit.

**Figure 3:**
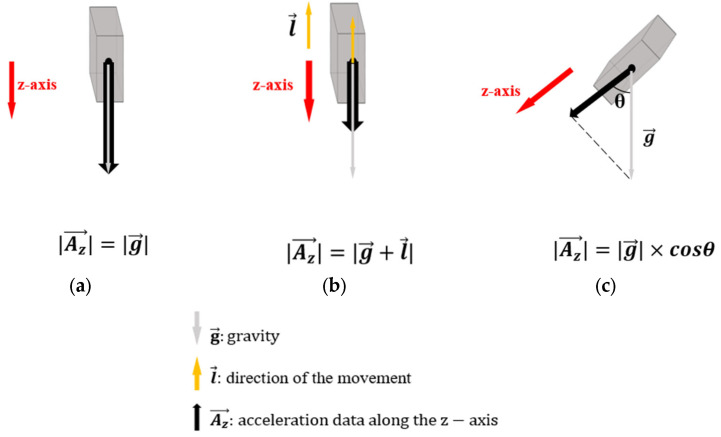
Direction and amplitude of the acceleration vector along the *z*-axis when (**a**) the *z*-axis is toward the ground and there is no movement, (**b**) the *z*-axis is toward the ground and the movement is in the opposite direction, (**c**) the *z*-axis direction together with the gravity creates an angle of θ ≠ 0 and there is no movement.

Figure 3 indicates the directions and amplitudes of the acceleration vector along the *z*-axis in three cases. In general, the acceleration vector is defined by gravity and a vector of the movement, and its amplitude is calculated by the following equation:

(1) $$\left|\vec{A_{z}}|=||\vec{g}+\vec{l}|\times cos\theta \right|$$

where $\vec{A_{z}}$ is the acceleration vector along the *z*-axis, $\vec{g}$ is gravity, $\vec{l}$ indicates the movement of the user’s arm that the IMU sensor is on, and θ is the angle created by $\vec{A_{z}}$ and $\vec{g}$.

In the first case (Figure 3a), $\vec{l}$ is a zero vector due to no movement; the angle θ is zero because vector $\vec{A_{z}}$ has the same direction as gravity. Therefore, $\vec{A_{z}}$ has an amplitude equal to Earth’s gravitational constant (G), which is approximately 9.8 m/s^2^. When the user makes a movement whose direction creates a straight angle with gravity (Figure 3b), the amplitude of $\vec{A_{z}}$ is equivalent to the vector sum of the vector of the movement and gravity.

(2) $$\left|\vec{A_{z}}|=|\vec{g}+\vec{l}\right|$$

Lastly, if the IMU sensor is worn so that the angle formed by the *z*-axis and the direction of gravity ranges between 0 degrees and 180 degrees (as illustrated in Figure 3c) and the sensor is in a stationary state ($\vec{l}$ = $\vec{0}$), the amplitude of $\vec{A_{z}}$ is calculated by multiplying G with the cosine of θ.

(3) $$\left|\vec{A_{z}}\right|=\left|\vec{g}\right|\times cos\theta$$

### 2.2. Data Acquisition and Preprocessing

**Figure 4:**
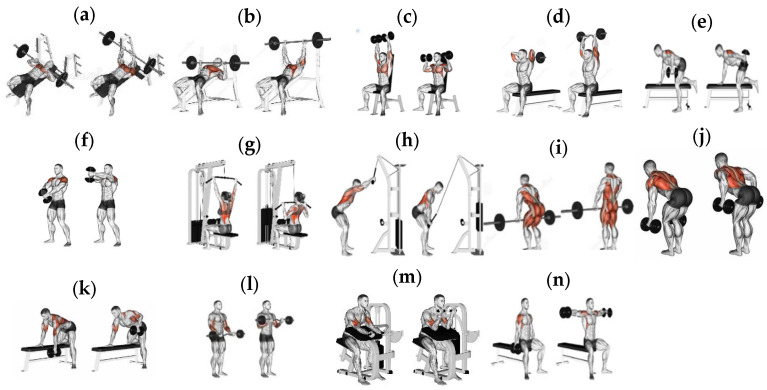
The 14 fitness exercises chosen to be classified. (**a**) Bench press, (**b**) Incline bench press, (**c**) Dumbbell shoulder press, (**d**) Dumbbell triceps extension, (**e**) Dumbbell kickback, (**f**) Dumbbell front raise, (**g**) Lat pull down, (**h**) Straight arm lat pull down, (**i**) Deadlift, (**j**) Dumbbell bent row, (**k**) One-arm dumbbell row, (**l**) EZ-bar curls, (**m**) Machine preacher curl, (**n**) Seated dumbbell lateral raise.

In this study, five participants were asked to wear a wristband with an IMU attached to it. With the IMU on the right wrist, each participant performed 14 exercises, which are illustrated in Figure 4.

**Figure 5:**
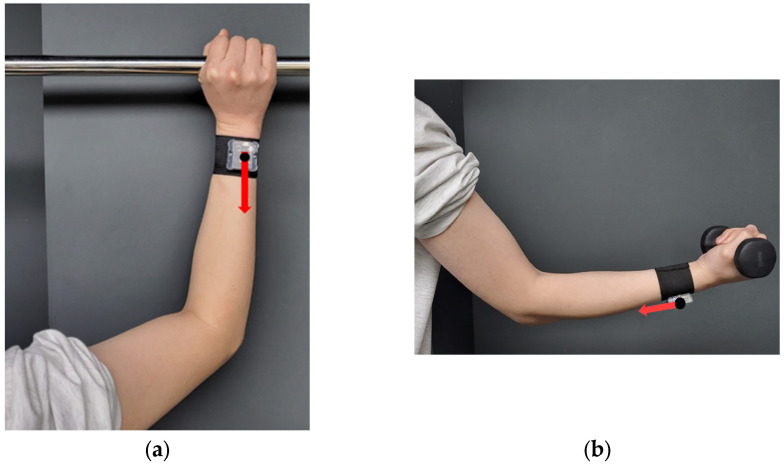

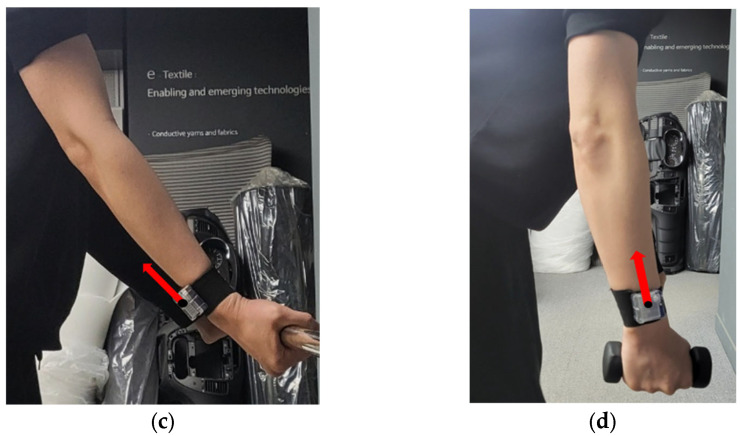
The direction of the *z*-axis of the accelerometer in four types of user’s forearm positions. (**a**) The *z*-axis has the same direction as gravity, (**b**) the *z*-axis is perpendicular to gravity, (**c**) the *z*-axis forms an angle with gravity larger than 90° but less than 180°, (**d**) the *z*-axis forms an angle of 180° with gravity. Red arrow indicates the direction of the *z*-axis.

These exercises were chosen based on the initial positions of the users’ forearms, belonging to one of the four depicted in Figure 5. The data acquisition procedure for each participant consisted of two phases. Firstly, they were asked to initiate each exercise with the position on the left, as demonstrated in Figure 4, for 5 s. Next, they performed the exercises according to the instructions in the figure for 10 cycles, which took approximately 20 s.

Due to the low mass of the IMU, which typically weighs around 15 g, any external force acting on the IMU would cause a significant alteration in the measured acceleration and introduce high-frequency noise to the signal [26]. Applying a low-pass filter with a cutoff frequency of 40 Hz can effectively remove high-frequency noise and preserve the physiological signal. The gravitational forces experienced during rest were assumed to be G.

### 2.3. Model Architecture

**Figure 6:**
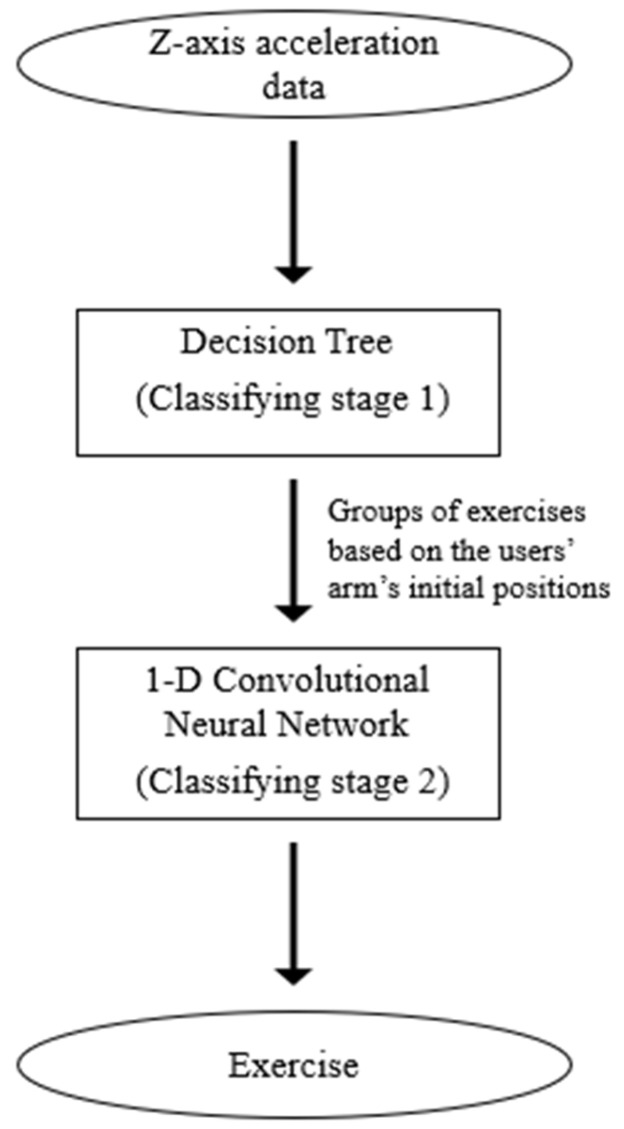
Block diagram of the proposed model with two classifying stages.

Figure 6 presents a block diagram of the proposed model to classify the fourteen fitness exercises.

The architecture of this model consists of two classifying stages. The first stage employed a decision tree to split the exercises into three or four groups based on the participant’s initial forearm position in each of them, which is mentioned in the previous section. The two methods to divide these exercises are shown in Table 1.

Specifically, in exercises (a), (b), (c), and (g), the participants started with their forearms perpendicular to the ground plane, so that the direction of the *z*-axis in the IMU acceleration sensor was identical to that of gravity. To perform exercises (e), (f), (i), (j), (k), (l), and (n), the participants initiated their forearms in a position so that the *z*-axis created an angle of either greater than 90° (exercises (f), (l)) or 180° (the rest) with gravity. Consequently, they were clustered into two groups, as in Method 2, or gathered in one group, as in Method 1. Lastly, if the angle constructed by the *z*-axis acceleration sensor and the direction of gravity was less than 90°, the participant’s forearms would be in the initial position of one of the following exercises: (d), (h), or (m). Therefore, they formed another group. Based on these characteristics, the decision tree, with the advantage of less effort required during preprocessing data compared to other algorithms, was employed as the first classifying stage of the proposed network.

**Figure 7:**
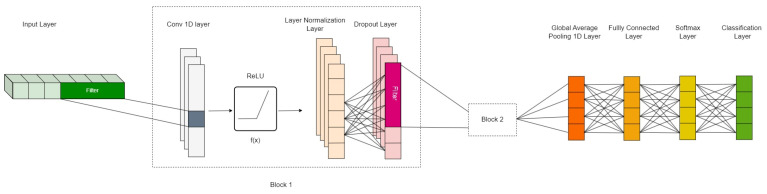
The 1D-CNN architecture—the second stage of the proposed model. The input is only one time series. The units of the output are the number of classes corresponding to each group. Block 1 and Block 2 have the same layers and activation function, while Block 1 has 23 filters for the Conv 1D layer and Block 2 has 46 filters for this layer.

The output of this stage, which is the *z*-axis acceleration data clustered in groups, was then passed to the second stage—a 1D-CNN [27]. The classification task for time-series data can be performed by utilizing either convolutional neural networks or recurrent networks. However, 1-D convolutional layers process the input with a single operation, therefore taking less time to learn features compared to recurrent layers that iterate over the time steps of the input [28]. Additionally, 1D-CNNs have been extensively deployed in classification models for sequences because of their advantages over 2D-CNNs, for instance, less-complicated configurations and hyperparameters, shallower architectures that make them easier to implement, smaller hardware setup, and low cost [29,30,31,32]. The 1D-CNN architecture employed in this research is illustrated in Figure 7.

The input layer of this stage receives the time series that is output from the first stage. Overall, this stage consists of two similar blocks (Block 1 and Block 2 in Figure 7); each block has a 1D convolution layer, a rectified linear unit (ReLU) activation function, a layer normalization layer, and a dropout layer. To learn the features of the input, the 1D convolution layer is employed, which has 23 filters with a size of 4. These filters perform convolutions in sequence, and the sum of these operations is the input of the activation function. This function assesses the effect of the input to each node and decides to activate the nodes with high contribution to the network. The ReLU output is normalized by the layer normalization layer so that its mean remains close to 0 and its standard deviation is close to 1. The limited dataset may cause overfitting. To reduce this effect, a dropout layer with a dropout rate of 0.5 is used. Specifically, 50% of neurons of the layer are randomly nullified or have their inputs set to 0, while to retain the sum of the inputs, the rest of them are scaled up. In the second block of this stage, the parameters of each layer are kept the same, only the number of filters in the 1D convolution layer increases twice to 46 filters, while the size of each filter remains unchanged. This aims to help the 1D-CNN layer learn more features from the input. After two blocks, the output of the second dropout layer is transformed to a 1D vector by a global average pooling 1D layer. The output of this layer is mapped to a vector of distinct probabilities by a fully connected layer with a size equal to the number of classes. This is followed by a softmax layer, which applies the normalized exponential function to the input, so it ranges between 0 and 1 and demonstrates the probabilities.

The whole cascaded model was defined in MATLAB software (R2021b), while the 1D-CNN stage was trained and fine-tuned using the Adam optimizer, with a learning rate of 0.01 for 200 epochs.

## 3. Results

To evaluate the efficiency of the proposed model, the authors compared the performance of this cascaded classifier and the 1D convolutional neural network on the same dataset.

### 3.1. Evaluation of 1D Convolutional Neural Network

**Figure 8:**
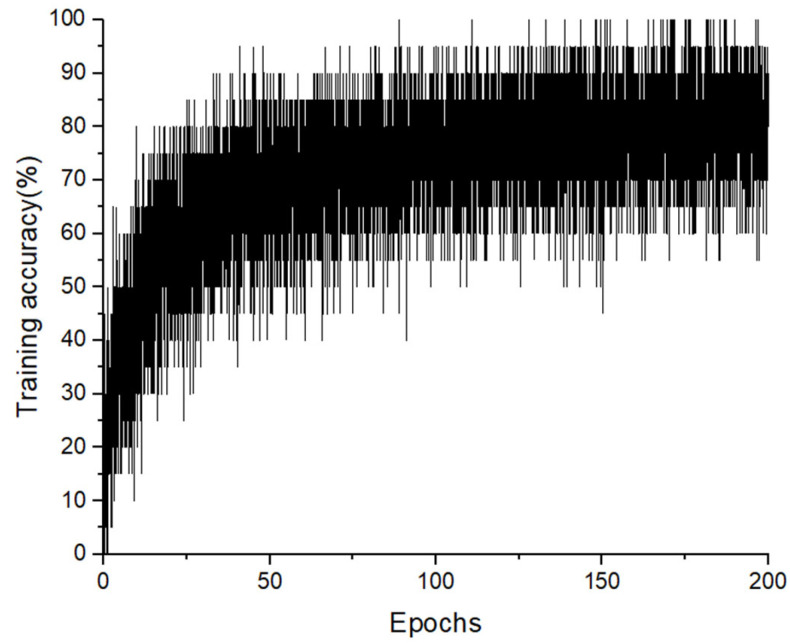
Training accuracy of the 1D convolutional neural network for 200 epochs with 14 classes.

Figure 8 presents the training accuracy of the 1D-CNN model. After 200 epochs, the model achieved a training accuracy of 80% and a test accuracy of 82.38%.

**Figure 9:**
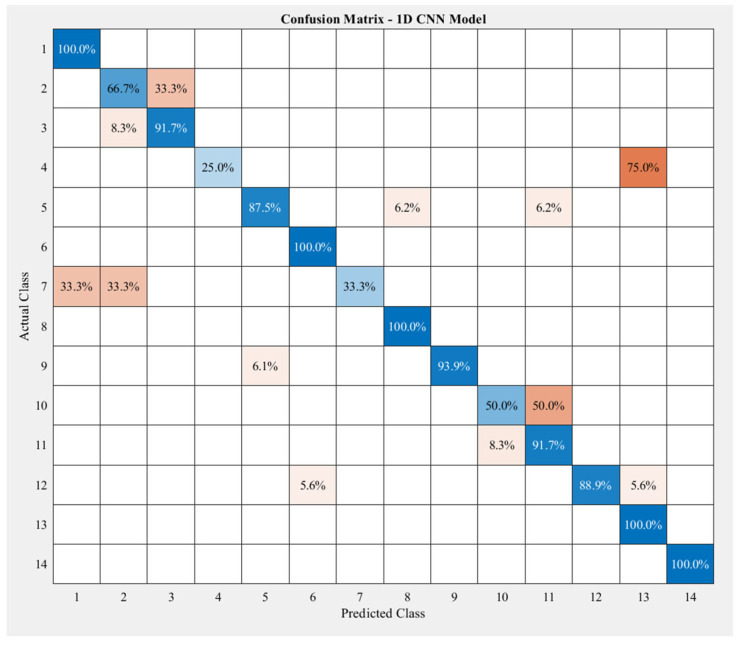
Accuracy of the 1D-CNN model on the test data with 14 classes. The order of the exercises in this confusion matrix is the same as that in Figure 4. The darker the blue, the higher the accuracy, while the darker the red, the lower the accuracy.

The confusion matrix in Figure 9 shows the correct prediction rates of this model on the test data with 14 labels.

As can be seen from the above matrix, the 1D-CNN classification model obtained the highest accuracy of 100% for five exercises, (a), (f), (h), (m), and (n), and the lowest accuracy of 25% and 33.3% for two exercises, (d) and (g). The classification accuracies for the rest of the exercises ranged between 50% and 93.9%. The highest misclassification rate was for the pair of two exercises (d) and (n); up to three-quarters of exercise (d) was misclassified as exercise (n), followed by the pair of exercises (j) and (k), which was 50%. In addition, the model performed poorly on classifying exercise (g); the rate was correct only 33.3% of the time. Other than that, it was clustered as exercise (a) (33.3%) and exercise (b) (33.3%).

### 3.2. Evaluation of the Cascaded Classifier

In this section, the results of the proposed cascaded classifier are presented. To investigate the impact of the decision tree classifying stage on the accuracy of the whole model, the authors ran the cascaded model in two cases that corresponded to the methods explained in Table 1.

#### 3.2.1. Method 1—All the Exercises Are Split into 3 Groups

**Figure 10:**
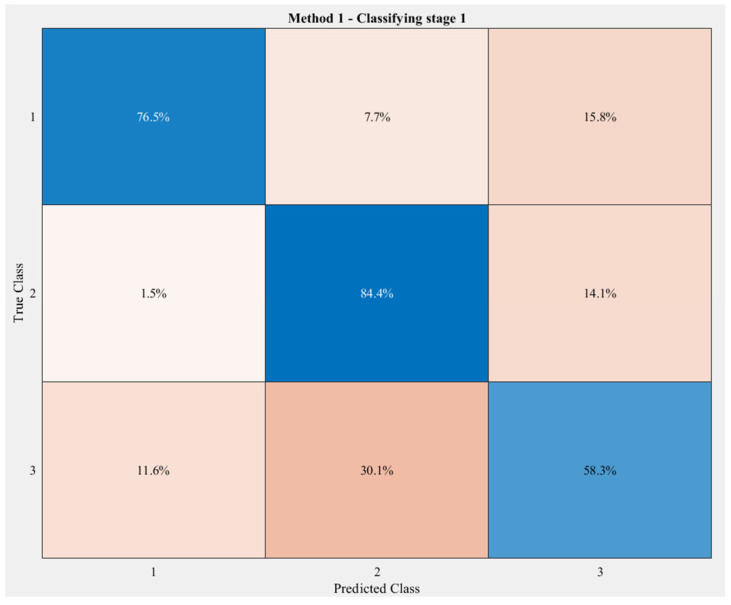
Classification results of the first stage with 14 exercises divided into three groups. The darker the blue, the higher the accuracy, while the darker the red, the lower the accuracy.

Figure 10 demonstrates the output of the first classifying stage with three classes corresponding to three groups, with an overall accuracy of 77.8%. Specifically, in this stage Group 2, which consists of 7 exercises, was well-classified the most, with a correct rate of 84.4%. Only 1.5% was misclassified as Group 1; this figure was relatively higher with Group 3—14.1%. Following Group 2, Group 1 had a slightly lower accuracy rate—76.5%. To be specific, 15.8% of this group was labeled as Group 3, but only half of this number (7.7%) was labeled as Group 2. Group 3 had the lowest correctly classified rate at approximately 58.3%, while up to one-third of the data was misclassified as Group 2 and roughly one-tenth as Group 1.

The correctly labeled data, which are the output of stage 1, were used for training and testing the stage 2 1D-CNN.

**Figure 11:**
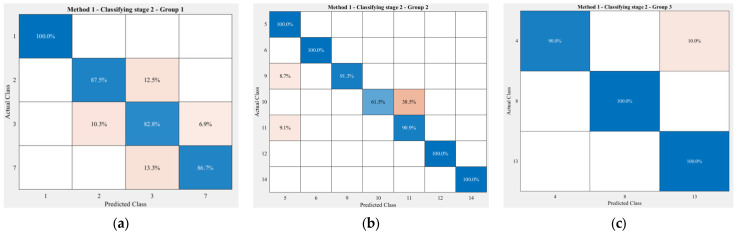
Classification results of the second stage for the exercises in Group 1 (**a**), Group 2 (**b**), and Group 3 (**c**) performed by Method 1. The order of the exercises in these confusion matrices is the same as that in Figure 4. The darker the blue, the higher the accuracy, while the darker the red, the lower the accuracy.

Figure 11 describes the performance of classifying stage 2 of the proposed cascaded model on three groups, with an overall accuracy of 92.49% on test data, which is around 10% higher than for the 1D convolutional neural network model. It is perceptible that the correctly classified rates for the 14 exercises achieved by the proposed model mainly varied from 82.8% to 100%, and up to seven exercises were labeled precisely by the model, namely exercises (a), (e), (f), (h), (l), (m) and (n). This second classifying stage did not perform well on clustering two exercises—(j) and (k). Specifically, around 38% of exercise (j) was misclassified, while this rate for the 1D-CNN model was exactly 50%. Other than that, on average, only approximately 10% of the other 13 exercises were clustered incorrectly by the model.

#### 3.2.2. Method 2—All the Exercises Are Split into 4 Groups

As was explained in the Section 2, in Method 2 all the exercises were split into four groups according to their difference in the angle θ constructed by the direction of the *z*-axis of the acceleration IMU sensor and the direction of gravity. To examine the necessity of dividing Group 2 in Method 1 into two subgroups, the authors assessed the accuracy of the whole cascaded model, with the results presented as follows.

**Figure 12:**
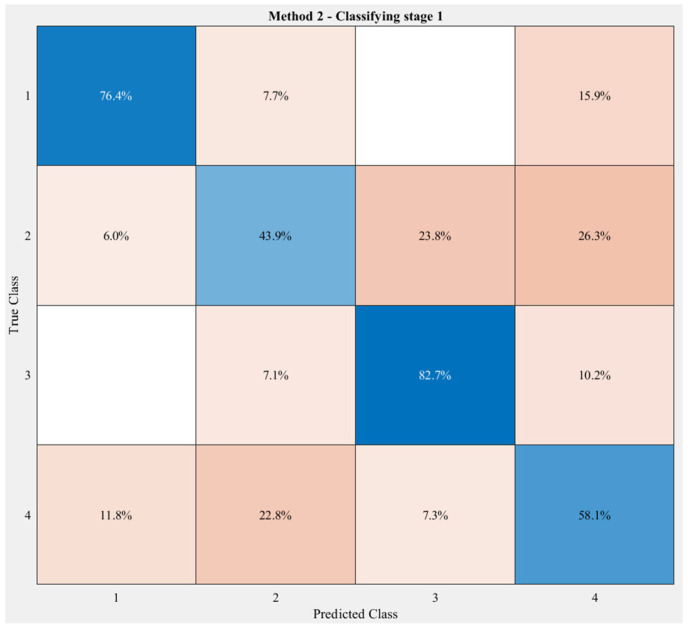
Classification results of the first stage, with 14 exercises divided into 4 groups. The darker the blue, the higher the accuracy, while the darker the red, the lower the accuracy.

Figure 12 illustrates the accuracy of the first classifying stage—decision tree—when applying Method 2. This number was slightly lower compared to Method 1, 72.2%. The results show that Group 2 was misclassified the most, with 26.3% of this group clustered in Group 4 and 23.8% in Group 3. A similar number was seen in the misclassification rate of Group 4 as of Group 2—22.8%.

**Figure 13:**
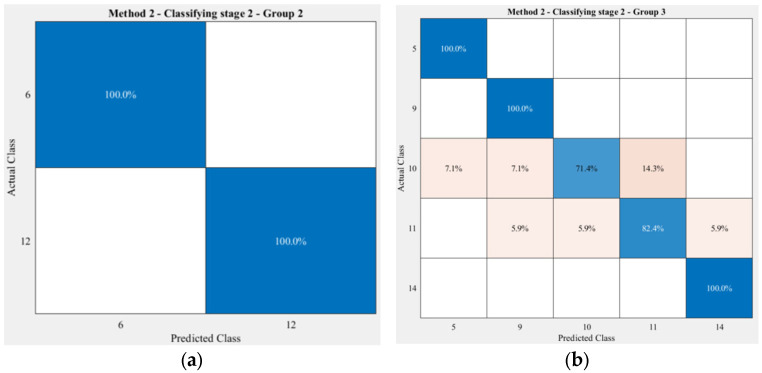
Classification results of the second stage for the exercises in Group 2 (**a**) and Group 3 (**b**) that were formed by Method 2. The order of the exercises in this confusion matrix is the same as that in Figure 4. The darker the blue, the higher the accuracy, while the darker the red, the lower the accuracy.

The only difference between the two methods is the separation of Group 2 in Method 1 into Group 2 and Group 3 in Method 2. Therefore, the accuracies achieved on the classification of exercises in Group 1 and Group 3 in Method 1 were identical to Group 1 and Group 4 in Method 2. Figure 13 indicates the performance of the second stage in the proposed cascaded model on two exercises in Group 2 and five exercises in Group 3.

From the obtained results, it is noticeable that the two exercises (f) and (l) were precisely classified in both methods. This 100% accuracy was also seen in the classification of exercises (e), (i), and (n). As with Method 1, in Method 2 the accuracy of exercise (j) was the lowest in comparison to the rest of the exercises, which was 71.4%. However, the model only misclassified exercise (j) with exercise (k) in Method 1, while in this method, besides exercise (k), the model clustered exercise (j) incorrectly as exercise (e) as well as exercise (j) at the same amount of 7.1%.

As a whole, the results show that the proposed cascaded model including two classifying stages of a decision tree and a 1D-CNN achieved a higher performance as compared with the 1D-CNN model on the same dataset, with similar training time. Between the two methods of clustering the exercises for the first stage of the model, Method 2 gave a better accuracy for each exercise.

## 4. Discussion

In classification problems, a decision tree is a useful machine learning algorithm due to its advantages. First, it does not require any transformation of the features. Compared to other classification algorithms such as KNN, it is very fast and efficient, and easy to understand and interpret. On top of that, decision tree models can handle various types of data with less data preparation needed. However, when the tasks come to time series examination, one-dimension convolutional neural networks (1D-CNNs) have gained a lot of attention and have been widely adopted [22,27,33,34,35]. Instead of applying some sort of noise filtering or smoothing techniques before the analysis, convolutional neural networks provide an effective way to learn the smoothing parameters. This comes from the architecture of the network, where the first two layers are generally a convolutional layer and a pooling layer, which both execute smoothing. Therefore, 1D-CNN models can learn from the raw time series data directly and do not require any manually engineered features as input. Alternatively, they learn to extract features from the sequences of observations and how to map the inner features to the classes.

Based on the superiority of these two models, the authors proposed a cascaded classifier that combines a decision tree as the first clustering stage and a 1D-CNN as the second classifying stage to classify 14 fitness exercises with the *z*-axis acceleration data acquired by an IMU sensor. From the results presented in the previous section, it is noticeable that the cascaded model performs better than the conventional 1D convolutional neural network. The training accuracy graph in Figure 7 also indicates a common problem of any machine learning models, which is the insufficiency of raw data. A lack in size or diversity of observations may cause the model to not have enough information to learn the latent patterns in the data, consequently leading to a large fluctuation in training/validating accuracy. In the range of this study, because the dataset was limited, the authors employed a decision tree as the first classifying stage because of its good performance even when the size of the dataset is small. This stage also helps to reduce the number of learnable parameters of the second stage, allowing the model to learn the hierarchical feature structure and reduce redundant features. Generally, the proposed cascaded model achieved an increase of approximately 10% in the overall test accuracy, in comparison with the 1D-CNN model.

In this study, the authors also presented two methods of clustering the exercises for the first stage of the model, which are based on the angle constructed by two vectors: the gravitational vector and the direction of the *z*-axis acceleration IMU sensor worn on the user’s wrist while they perform the exercises. A set of 14 exercises were divided into three groups in Method 1 and four groups in Method 2. Although in the first method, Group 2 and Group 3 from Method 2 were gathered, the confusion matrix in Figure 12 shows that the Group 2 formed by the second method is more like Group 4. This result partly helps to inspect the similarity of the chosen exercises and come to a more efficient clustering decision for them in future work. In terms of the efficiency of the first classifying stage in both methods, Method 1 provided a slightly better result, 77.8% compared to 72.2% for Method 2. However, the two methods showed similar accuracy after the second classifying stage, which was around 92%. While Method 1 misclassified almost 40% of exercise (j) as exercise (k), this rate was reduced to only 14.3% in Method 2. Therefore, the correctly classified rate of each exercise in Method 2 was higher than in Method 1. In general, the cascaded model achieved a higher accuracy than that of the conventional 1D-CNN models. The examination of two clustering methods indicates that the first classifying stage—decision tree—in the proposed cascaded model greatly contributes to the performance of the whole model, though the average accuracies of the model in both methods were identical.

Nonetheless, this research still presents some limitations. First, the proposed model was only utilized to classify 14 forearm/upper body exercises, while the need for classifying a large number of fitness exercises will increase over time. To achieve good performance without increasing the time of acquiring and preprocessing data, the authors only exploited the *z*-axis acceleration data of the IMU sensor, which limited the possible classes of the first classifying stage and led to its misclassification of some exercises.

In future work, the authors expect to increase the number of exercises that can be classified. This work involves the addition of the *x*-axis and *y*-axis acceleration data from the IMU sensor to expand the latent features of the dataset. Along with the increase in the amount of data, the authors continue to develop and optimize the first classifying stage to enhance its accuracy while minimizing the training time, data preparation, and data preprocessing process, which are crucial criteria for real-time application.

## 5. Conclusions

In this study, the authors proposed an architecture for a cascaded model to classify 14 upper-body exercises, using the *z*-axis acceleration data from an IMU sensor. The results obtained in this research demonstrated that (1) only data measured along the *z*-axis of the accelerometer in the IMU sensor are needed for the task of classifying fitness exercises, instead of six-axis or nine-axis data; and (2) a cascaded model consisting of two classifying stages can effectively classify time series data with an increase in accuracy and the same training time, compared to the conventional 1D-CNN models. Further studies need to be performed in order to enhance the performance of each stage in the model and, additionally, expand the diversity of the input data and hence aim to classify a larger amount of fitness exercises.

## Figures and Tables

**Table 1:** Two methods to cluster all the exercises into classes for the first classifying stage.

Method 1: Exercises are split into three groups				
	Group 1	Group 2		Group 3
Forearm’s initial position	(a) *	(c), (d)		(b)
Exercise	(a), (b), (c), (g) **	(e), (f), (i), (j), (k), (l), (n)		(d), (h), (m)
Method 2: Exercises are split into four groups				
	Group 1	Group 2	Group 3	Group 4
Forearm’s initial position	(a)	(c)	(d)	(b)
Exercise	(a), (b), (c), (g)	(f), (l)	(e), (i), (j), (k), (n)	(d), (h), (m)

* The labels of the forearm’s initial position follow those in Figure 5. ** The exercises’ labels follow those in Figure 4.

## Data Availability

Not applicable.

## References

[1] Gupta N., Gupta S.K., Pathak R.K., Jain V., Rashidi P., Suri J.S. (2022). Human Activity Recognition in Artificial Intelligence Framework: A Narrative Review. Artif. Intell. Rev..

[2] Yadav S.K., Tiwari K., Pandey H.M., Akbar S.A. (2021). A Review of Multimodal Human Activity Recognition with Special Emphasis on Classification, Applications, Challenges and Future Directions. Knowl. Based Syst..

[3] Sunny J.T., George S.M. (2015). Applications and Challenges of Human Activity Recognition Using Sensors in a Smart Environment. Int. J. Innov. Res. Sci. Technol.

[4] Ranasinghe S., Al Machot F., Mayr H.C. (2016). A Review on Applications of Activity Recognition Systems with Regard to Performance and Evaluation. Int. J. Distrib. Sens. Netw..

[5] Zhu R., Xiao Z., Li Y., Yang M., Tan Y., Zhou L., Lin S., Wen H. (2019). Efficient Human Activity Recognition Solving the Confusing Activities Via Deep Ensemble Learning. IEEE Access.

[6] Mokari M., Mohammadzade H., Ghojogh B. (2020). Recognizing Involuntary Actions from 3D Skeleton Data Using Body States. Sci. Iran..

[7] Zhao Y., Zhou H., Lu S., Liu Y., An X., Liu Q. (2022). Human Activity Recognition Based on Non-Contact Radar Data and Improved PCA Method. Appl. Sci..

[8] Vu C.C., Kim J. (2018). Human Motion Recognition by Textile Sensors Based on Machine Learning Algorithms. Sensors.

[9] Fan Y.-C., Tseng Y.-H., Wen C.-Y. (2022). A Novel Deep Neural Network Method for HAR-Based Team Training Using Body-Worn Inertial Sensors. Sensors.

[10] Nyenhuis S.M., Greiwe J., Zeiger J.S., Nanda A., Cooke A. (2020). Exercise and Fitness in the Age of Social Distancing During the COVID-19 Pandemic. J. Allergy Clin. Immunol. Pract..

[11] Hurley O.A. Sport Cyberpsychology in Action During the COVID-19 Pandemic (Opportunities, Challenges, and Future Possibilities): A Narrative Review. https://pubmed.ncbi.nlm.nih.gov/33746838/.

[12] Keshkar S., Karegar G.A. (2022). Effect of the COVID-19 Pandemic on the Sports Industry. COVID-19 and the Sustainable Development Goals.

[13] Prakash J., Yang Z., Wei Y.-L., Choudhury R.R. (2019). STEAR: Robust Step Counting from Earables. Proceedings of the 1st International Workshop on Earable Computing.

[14] Wahjudi F., Lin F.J. IMU-Based Walking Workouts Recognition. Proceedings of the 2019 IEEE 5th World Forum on Internet of Things (WF-IoT).

[15] Hausberger P., Fernbach A., Kastner W. IMU-Based Smart Fitness Devices for Weight Training. Proceedings of the IECON 2016-42nd Annual Conference of the IEEE Industrial Electronics Society.

[16] ACM Digital Library Wearable IMU-Based Gym Exercise Recognition Using Data Fusion Methods. https://dl.acm.org/doi/10.1145/3469678.3469705.

[17] Biswas D., Cranny A., Gupta N., Maharatna K., Achner J., Klemke J., Jöbges M., Ortmann S. (2015). Recognizing Upper Limb Movements with Wrist Worn Inertial Sensors Using K-Means Clustering Classification. Hum. Mov. Sci..

[18] Balfany K. (2019). A Practical Method to Monitor Muscle Activation as an Indicator of Training Load During Fatiguing Exercise.

[19] Yasuda T., Brechue W.F., Fujita T., Shirakawa J., Sato Y., Abe T. (2009). Muscle Activation during Low-Intensity Muscle Contractions with Restricted Blood Flow. J. Sports Sci..

[20] Li C., Liu D., Xu C., Wang Z., Shu S., Sun Z., Tang W., Wang Z.L. (2021). Sensing of Joint and Spinal Bending or Stretching via a Retractable and Wearable Badge Reel. Nat. Commun..

[21] Saucier D.N., Davarzani S., Burch V.R.F., Chander H., Strawderman L., Freeman C., Ogden L., Petway A., Duvall A., Crane C. (2021). External Load and Muscle Activation Monitoring of NCAA Division I Basketball Team Using Smart Compression Shorts. Sensors.

[22] Mattioli F., Porcaro C., Baldassarre G. (2022). A 1D CNN for High Accuracy Classification and Transfer Learning in Motor Imagery EEG-Based Brain-Computer Interface. J. Neural. Eng..

[23] Li F., Liu M., Zhao Y., Kong L., Dong L., Liu X., Hui M. (2019). Feature Extraction and Classification of Heart Sound Using 1D Convolutional Neural Networks. EURASIP J. Adv. Signal Process..

[24] Shahid S.M., Ko S., Kwon S. Performance Comparison of 1D and 2D Convolutional Neural Networks for Real-Time Classification of Time Series Sensor Data. Proceedings of the 2022 International Conference on Information Networking (ICOIN).

[25] Burns D., Leung N., Hardisty M., Whyne C., Henry P., McLachlin S. (2018). Shoulder Physiotherapy Exercise Recognition: Machine Learning the Inertial Signals from a Smartwatch. Physiol. Meas..

[26] Day E.M., Alcantara R.S., McGeehan M.A., Grabowski A.M., Hahn M.E. (2021). Low-Pass Filter Cutoff Frequency Affects Sacral-Mounted Inertial Measurement Unit Estimations of Peak Vertical Ground Reaction Force and Contact Time during Treadmill Running. J. Biomech..

[27] Brownlee J. (2018). 1D Convolutional Neural Network Models for Human Activity Recognition. https://machinelearningmastery.com/cnn-models-for-human-activity-recognition-time-series-classification.

[28] LeCun Y., Bengio Y., Hinton G. (2015). Deep Learning. Nature.

[29] Kiranyaz S., Ince T., Hamila R., Gabbouj M. Convolutional Neural Networks for Patient-Specific ECG Classification. Proceedings of the 37th Annual International Conference of the IEEE Engineering in Medicine and Biology Society (EMBC).

[30] Kiranyaz S., Gastli A., Ben-Brahim L., Al-Emadi N., Gabbouj M. (2019). Real-Time Fault Detection and Identification for MMC Using 1-D Convolutional Neural Networks. IEEE Trans. Ind. Electron..

[31] Abdeljaber O., Sassi S., Avci O., Kiranyaz S., Ibrahim A.A., Gabbouj M. (2019). Fault Detection and Severity Identification of Ball Bearings by Online Condition Monitoring. IEEE Trans. Ind. Electron..

[32] Eren L., Ince T., Kiranyaz S. (2019). A Generic Intelligent Bearing Fault Diagnosis System Using Compact Adaptive 1D CNN Classifier. J. Signal Process Syst..

[33] Sheng H., Liu M., Hu J., Li P., Peng Y., Yi Y. (2023). LA-ESN: A Novel Method for Time Series Classification. Information.

[34] Kuang D. (2020). A 1d Convolutional Network for Leaf and Time Series Classification. arXiv.

[35] Tang W., Long G., Liu L., Zhou T., Jiang J., Blumenstein M. (2020). Rethinking 1D-CNN for Time Series Classification: A Stronger Baseline. arXiv.

